# Human Regulatory T Cell Suppressive Function Is Independent of Apoptosis Induction in Activated Effector T Cells

**DOI:** 10.1371/journal.pone.0007183

**Published:** 2009-09-25

**Authors:** Yvonne Vercoulen, Ellen J. Wehrens, Nienke H. van Teijlingen, Wilco de Jager, Jeffrey M. Beekman, Berent J. Prakken

**Affiliations:** 1 Department of Pediatric Immunology, Center for Molecular and Cellular Intervention, Wilhelmina Children's Hospital, University Medical Center Utrecht, Utrecht, The Netherlands; 2 Molecular Immunology Lab, Department of Immunology, Wilhelmina Children's Hospital, University Medical Center Utrecht, Utrecht, The Netherlands; 3 Eureka Institute for Translational Medicine, Siracusa, Italy; New York University School of Medicine, United States of America

## Abstract

**Background:**

CD4^+^CD25^+^FOXP3^+^ Regulatory T cells (Treg) play a central role in the immune balance to prevent autoimmune disease. One outstanding question is how Tregs suppress effector immune responses in human. Experiments in mice demonstrated that Treg restrict effector T cell (Teff) responses by deprivation of the growth factor IL-2 through Treg consumption, resulting in apoptosis of Teff.

**Principal Findings:**

In this study we investigated the relevance of Teff apoptosis induction to human Treg function. To this end, we studied naturally occurring Treg (nTreg) from peripheral blood of healthy donors, and, to investigate Treg function in inflammation *in vivo*, Treg from synovial fluid of Juvenile Idiopathic Arthritis (JIA) patients (SF-Treg). Both nTreg and SF-Treg suppress Teff proliferation and cytokine production efficiently as predicted. However, in contrast with murine Treg, neither nTreg nor SF-Treg induce apoptosis in Teff. Furthermore, exogenously supplied IL-2 and IL-7 reverse suppression, but do not influence apoptosis of Teff.

**Significance:**

Our functional data here support that Treg are excellent clinical targets to counteract autoimmune diseases. For optimal functional outcome in human clinical trials, future work should focus on the ability of Treg to suppress proliferation and cytokine production of Teff, rather than induction of Teff apoptosis.

## Introduction

CD4^+^CD25^+^FOXP3^+^ regulatory T cells (Treg) are of critical importance for the maintenance of immune homeostasis, as numerous experimental mouse models for autoimmune diseases correlate the presence of functional Tregs with amelioration of disease severity [1], [2]. In humans Treg also play an important role in the immune balance, as patients lacking functional Treg, due to loss-of-function mutations in the transcription factor FOXP3, suffer from severe generalized autoimmune disease; immune dysregulation, polyendocrinopathy, enteropathy, X-linked (IPEX)[3], [4]. In addition, in human autoimmune diseases, like Juvenile Idiopathic Arthritis (JIA), negative correlations are found between the presence of regulatory T cells and disease severity[5]. Therefore, Treg are considered an important therapeutic target for a large range of human immune mediated diseases, and ongoing clinical trials attempt to modulate the population of Treg, and thereby restore immune balance. For example, in diabetes mellitus type 1, patients were treated with anti-CD3 antibodies in order to enhance Treg function, which resulted in clinical improvement and increased residual β-cell function[6], [7]. Moreover, in a clinical trial for cord blood transplantation in patients suffering from haematological cancer, infusion of donor-derived Treg is tested to prevent or reduce Graft versus Host Disease (GvHD) (NCT00602693, www.clinicaltrials.gov).

Despite these potentially far-reaching applications of Tregs in humans, questions remain with regard to the underlying mechanisms of Treg action, particularly in humans. Tregs may suppress effector cells either through cell-cell contact, the production of suppressive cytokines, and/or through the consumption of cytokines and growth factors such as IL-2 [8]. It is clear that IL-2 in many aspects is crucial for Treg function [9], [10]. For one, it is required for Treg expansion, and regulates FOXP3 expression [11], [12], and it is also indispensable for Treg mediated suppression [13]. On the other hand, FOXP3 suppresses IL-2 transcription, by binding to the IL-2 promoter [14], [15]. As a result Treg do not produce IL-2, and even may act as a ‘sink’ for IL-2. Thus, competition for IL-2 between effector T cells (Teff) and Treg, which express a higher level of IL-2Rα chain (CD25) compared to Teff, may counteract proliferation of Teff [16], [17]. Accordingly, Pandiyan et al. recently showed that in mice Treg consume IL-2 and thereby induce apoptosis in the Teff population [18], [19]. This mechanism of apoptosis through cytokine deprivation was responsible for the suppressive function of Treg. Consistently, IL-2 and other IL-2Rγ-chain binding cytokines, such as IL-7, were able to overcome cell death [18], and, in earlier reports, have been shown to interfere with both murine and human Treg-mediated suppression [17], [20].

We aimed to determine whether apoptosis induction via cytokine consumption by Treg is an important mechanism for human Treg-mediated suppression of Teff. As it is not fully understood how human Treg mediate their suppressive action on Teff, we studied the suppressive capacity and induction of apoptosis by naturally occurring Treg from peripheral blood and compared it to, assumedly *in vivo* activated Treg from an inflammatory site, the synovial fluid, of JIA patients. Our findings demonstrate that apoptosis induction in Teff is not important for human Treg mediated suppression.

## Results

### nTreg are highly suppressive without inducing apoptosis in Teff

**Figure 1:**
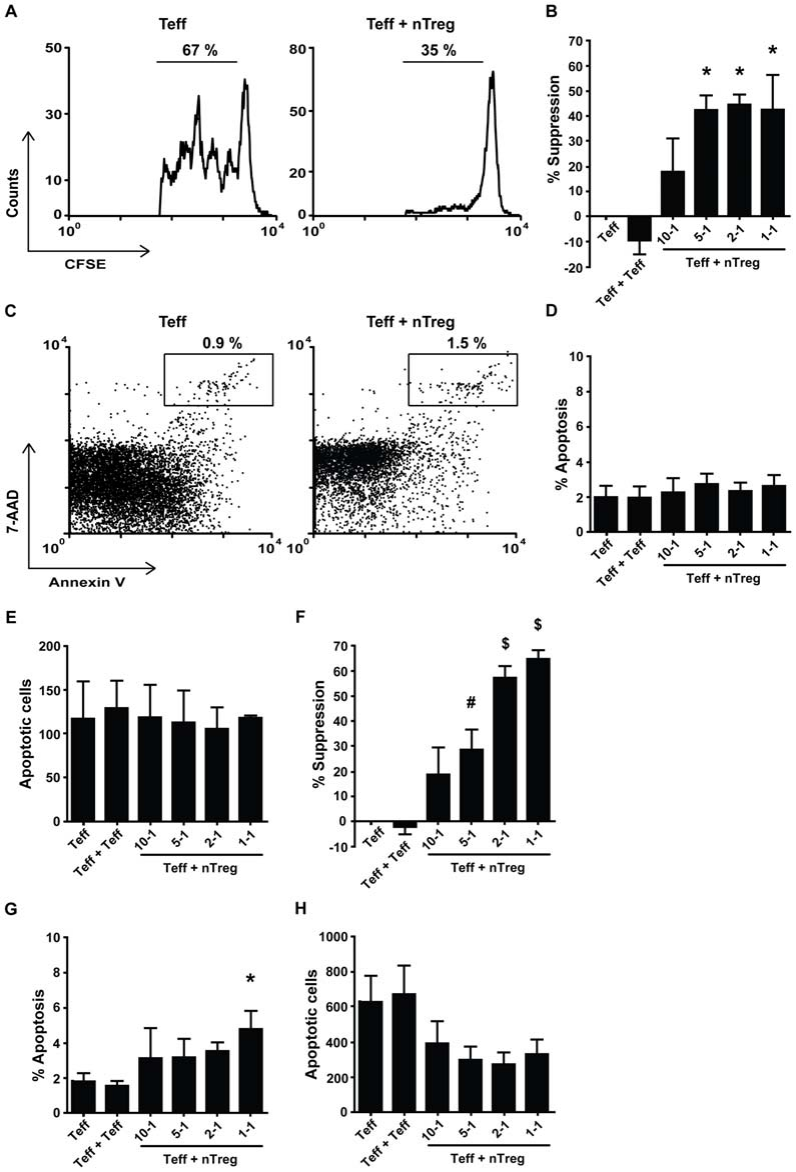
Naturally occurring Treg suppress Teff proliferation, but do not induce apoptosis. Cells were cultured in 200 µl medium for 5 days (n = 3). (A) Proliferation of Teff measured by flow cytometry, cultured alone (left) or in co-culture with naturally occurring Treg (1-1) (right). 1 representative example is shown. (B) Level of suppression of Teff proliferation, calculated for several ratios of Teff + Treg, and Teff + Teff (ratio 1-1), compared to culture of Teff alone (suppression = 0%). (C) Apoptotic Teff cells (CFSE^+^) were measured after 7-AAD and Annexin V staining by flow cytometry analysis. Percentage of apoptosis in Teff cultured alone (left) and in co-culture with Treg (1-1) (right). 1 representative example is shown. (D) Average percentage, and (E) absolute number, corrected for cell input, of apoptotic Teff expressing 7-AAD and Annexin V, for several co-culture ratios of Teff + Treg, Teff + Teff (1-1) and Teff alone,. Cells were cultured in 75 µl medium for 5 days (n = 9). (F) Level of suppression of Teff proliferation, calculated for several ratios of Teff + Treg, and Teff + Teff (ratio 1-1), compared to culture of Teff alone (suppression = 0%). (G) Average percentage, and (H) absolute number, corrected for cell input, of apoptotic Teff cells expressing 7-AAD and Annexin V, for several co-culture ratios of Teff + Treg, Teff + Teff and Teff alone (n = 9). Error bars represent means±s.e.m., * P<0.05, # P<0.01, ▒ P<0.001.

We first established that human Treg inhibit proliferation of activated Teff. CFSE labeled Teff were co-cultured for 5 days with a graded amount of CD4^+^CD25^+^CD127^low^ naturally occurring Treg (nTreg), in 200 µl culture medium, and suppression of Teff proliferation and induction of Teff apoptosis were determined. As expected, nTreg inhibited proliferation of Teff, as measured by decreased CFSE dilution in Teff cells (Figure 1A). This suppression of proliferation increased with titrated amounts of Treg in the culture, in a dose-dependent manner (Figure 1B).

Next, cells from the same co-cultures were stained with 7-AAD and Annexin V and gated on CFSE+ cells (See Figure S1A, B) to determine apoptosis in Teff. Only few apoptotic cells were found in cultures with Teff only, and the percentage of apoptotic cells did not increase upon the presence of more nTreg (Figure 1C, D), which was similar for the absolute number of apoptotic cells (Figure 1E). Thus under normal culture conditions, human nTreg do not induce apoptosis in Teff, while efficiently suppressing Teff proliferation.

We hypothesized that if cytokine consumption by Treg in the vicinity is responsible for apoptosis in Teff, culture of the same number of Teff and Treg in a smaller volume should enhance suppression mediated by apoptosis induction. Therefore, all further cultures were performed in 75 µl instead of 200 µl medium. Under these conditions the level of suppression was higher (up to 65% average at a 1-1 ratio) compared to normal culture conditions (up to 48% average at a 1-1 ratio) (Figure 1F). Furthermore, a larger number of Teff became apoptotic (up to 750 Annexin V^+^7-AAD^+^ cells average for Teff+Teff ) (Figure 1H), but in the co-cultures with nTreg the percentage of apoptotic cells only slightly increased (Figure 1G), and the number of apoptotic Teff even decreased (Figure 1H). Although we show a low upregulation of Annexin V on highly activated cells (Figure S3A), the level of apoptosis per cell division was independent of the presence of Treg (Figure S5). To establish that day 5 was the appropriate timepoint to measure apoptosis in our assays, we also measured cell death on day 3 and 4. Consistently, on day 3 and 4 hardly any apoptosis was seen (Figure S1C, D). Furthermore, we show that Teff in our assay are able to go into apoptosis, by titrating Sheath Fluid (BD Biosciences), containing ethanol into cultures with Teff (Figure S2A, B), causing Teff apoptosis in a dose-dependent manner. Thus, apoptosis induction does not occur in Teff + nTreg co-cultures, whereas high levels of suppression are reached. Altogether, these data clearly demonstrate that apoptosis induction is not important for nTreg mediated suppression.

### IL-2 and IL-7 overcome suppression, without influencing apoptosis

**Figure 2:**
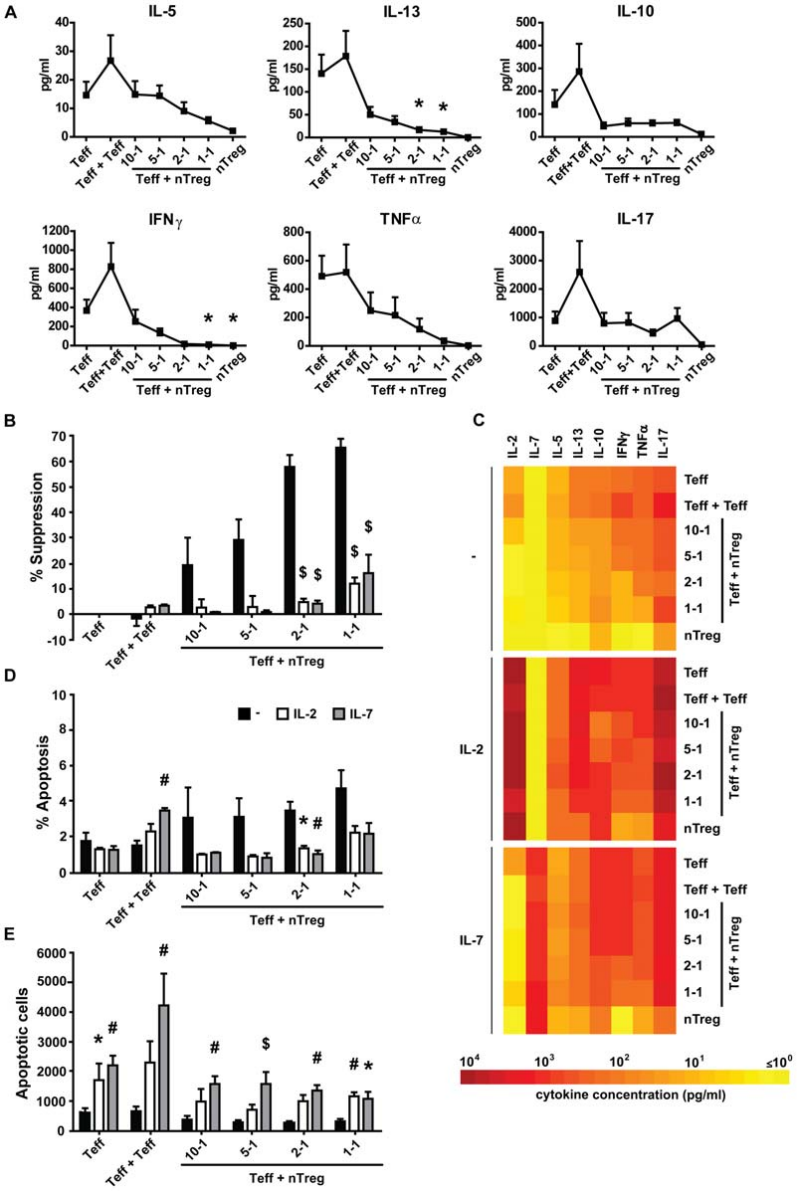
Exogenous IL-2 and IL-7 decrease suppression of Teff proliferation and cytokine production, but do not decrease apoptosis. Cells were cultured in 75 µl medium for 5 days (A) Levels of cytokines, present in culture medium, on day 5 of culture, in several co-culture ratios of Teff + Treg, Teff + Teff (1-1), and Teff alone (n = 9). (B) Level of suppression of CFSE^+^ Teff proliferation, calculated for several ratios of Teff + Treg, Teff + Teff (1-1) and Teff alone (suppression = 0%), cultured without (black bars), or with IL-2 (white bars) or IL-7 (grey bars). Suppression was calculated by comparing co-cultures with Teff alone with equal cell culture conditions. (C) Mean levels of cytokines, present in culture medium on day 5 of culture, in medium (-), with addition of IL-2 (IL-2) or IL-7 (IL-7). A color profile of the means was made to show the differences between culture conditions (see also Table S1). (D) Average percentage, and (E) absolute number, corrected for cell input,of apoptotic CFSE^+^ Teff cells, expressing 7-AAD and Annexin V, in several co-culture ratios of Teff + Treg and Teff alone, cultured without or with IL-2 or IL-7. (B–E, n = 5). Error bars represent means±s.e.m., * P<0.05, # P<0.01, ▒ P<0.001.

In mice, cytokine consumption was suggested to be pivotal for Treg-mediated apoptosis in Teff and suppression. Therefore, we investigated whether absence of IL-2Rγ-chain binding cytokines plays a role in the induction of apoptosis in Teff and suppression by human nTreg. In co-cultures of Teff and nTreg we observed a clear decrease in IL-2, as well as other cytokines important for Teff function; IL-5, IL-13, IL-10, IFNγ, TNFα, but not IL-17 (Figure 2A, C (upper panel)). This lack of IL-17 suppression could be due to a resistance of Th17 cells to Treg mediated suppression [21], [22] The decrease of cytokines in the culture medium in the presence of Treg could be due to either a general suppression of Teff cytokine production, or to cytokine consumption.

To investigate this further, we studied whether exogenously added cytokines could affect apoptosis induction of Teff, or suppression of proliferation and cytokine production by Teff. When high concentrations of exogenous IL-2 or IL-7 were added, the proliferation of Teff cells increased (data not shown). Furthermore, suppression of Teff proliferation was abrogated in all co-culture ratios (Figure 2B), which is in line with studies describing abrogation of Treg mediated suppression by IL-2 and IL-7, by either Teff stimulation, or, in case of IL-2, abrogation of Treg anergy [17], [20], [23]–[25]. The high levels of IL-2 or IL-7 abrogated nTreg-mediated suppression of cytokine production by Teff as well (Figure 2C and Table S1). Furthermore, it seems that both IL-2 and IL-7 increase cytokine production of Treg, which may have contributed to abrogation of suppression. In contrast, although the percentage of apoptotic cells seems to decrease (Figure 2D and Figure S3B), IL-2 and IL-7 did not decrease numbers of apoptotic Teff in co-cultures, instead the number of apoptotic cells was even significantly increased (Figure 2E) Thus, IL-2Rγ-chain binding cytokines prevent suppression of Teff proliferation and cytokine production, but this is not accompanied by a reduction in apoptosis. Although we cannot conclude from these data whether cytokine consumption is involved, this emphasizes that nTreg mediated suppression is independent of apoptosis induction in Teff.

### Apoptosis induction in Teff is irrelevant for Treg function in inflammation

**Figure 3:**
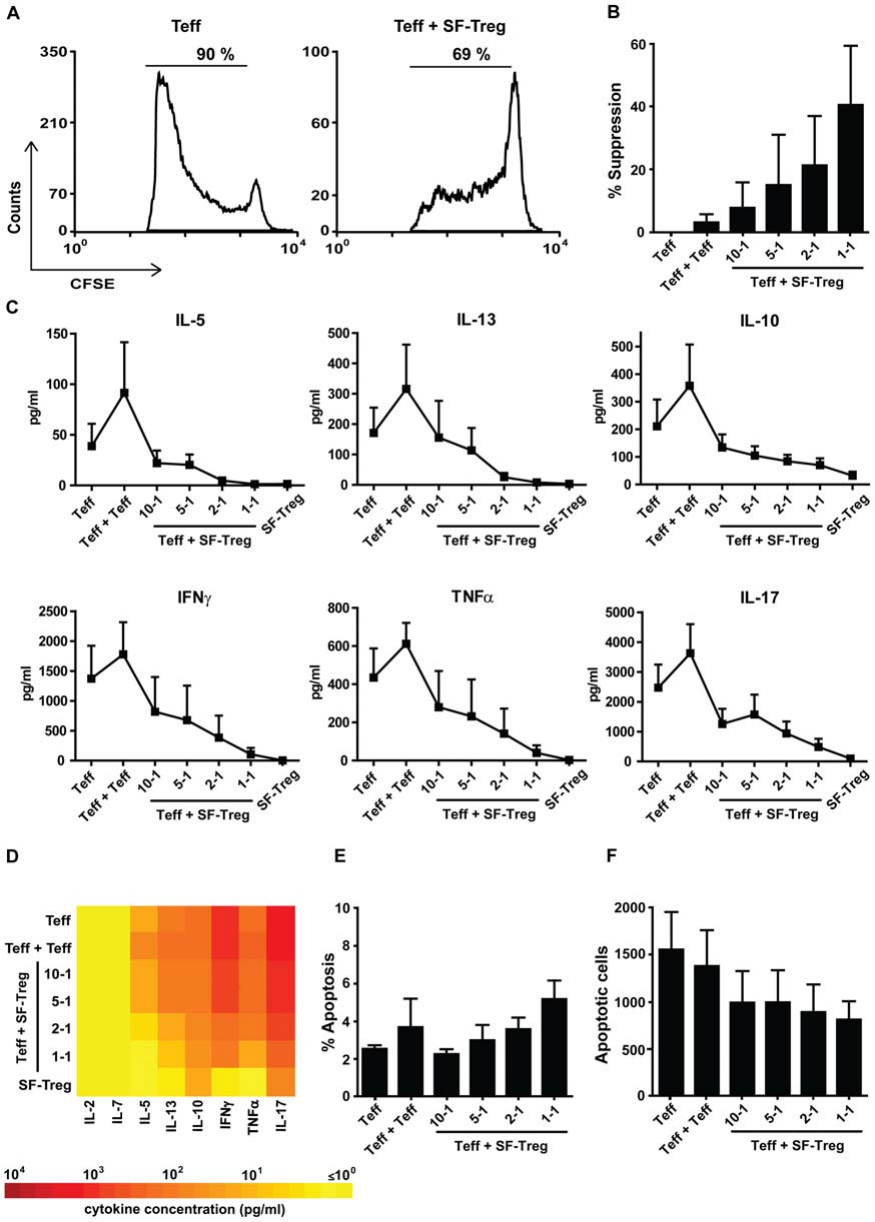
Synovial fluid-derived Treg suppress Teff proliferation and cytokine production, but do not induce apoptosis. Cells were cultured in 75 µl medium for 5 days. (A) Proliferation of CFSE^+^ Teff measured by flow cytometry, cultured alone (left) or in co-culture with SF-Treg (1-1) (right). 1 representative example is shown. (B) Level of suppression of CFSE^+^ Teff proliferation, calculated for several ratios of Teff + SF-Treg, and Teff + Teff (1-1), compared to culture of Teff alone (suppression = 0%). (C) Levels of cytokines, present in culture medium, on day 5 of culture, in several co-culture ratios of Teff + SF-Treg, Teff + Teff (1-1) and Teff alone. (D) Mean levels of cytokines, present in culture medium on day 5 of culture. A color profile of the means was made to show the differences between culture conditions (see also Table S2). (E) Average percentage, and, (F) absolute number, corrected for cell input, of apoptotic CFSE^+^ Teff for several co-culture ratios of Teff + SF-Treg, Teff + Teff (1-1) and Teff alone (B–E, n = 3). Error bars represent means±s.e.m.

Last, we wished to establish the relevance of our findings on nTreg to human Treg function in ongoing inflammation. Therefore, we studied Treg from within a chronically inflamed environment, the synovial fluid of JIA patients (SF-Treg). In synovial fluid, Treg are abundantly present and highly activated, due to the chronic inflammation. Furthermore, Teff from the SF probably have a different activation state, which may be contributing to the ongoing inflammation in JIA. To make a reliable comparison between Treg from the peripheral blood and Treg from the site of inflammation, it is preferable to use the same Teff population in all assays. Therefore, we co-cultured SF-Treg with Teff obtained from peripheral blood of the same patient in 75 µl medium. Probably due a different cellular composition, which may be caused by contaminating activated T cells, suppression of Teff proliferation by SF-Treg was less compared to nTreg (Figure 3A, B), whereas suppression of Teff cytokine production was similar to nTreg (Figure 3C, D, see also Tables S2 and S3). Still, similar as for nTreg, despite a slight increase of the percentage of apoptotic cells, a decreased number of apoptotic Teff was found in the presence of SF-Treg (Figure 3E, F). Altogether, even Treg from an inflammatory environment do not induce apoptosis in Teff cells to achieve suppression of Teff proliferation and cytokine production.

## Discussion

Pivotal studies in mice models have pointed out that Treg are indispensable for the maintenance of peripheral immune tolerance. Also in humans a similar role of Tregs is likely, prompting discussions about their clinical applicability. Though comparable in many aspects, several differences between mouse and human Treg phenotype, function and mechanisms of suppression have been identified in the past few years. For instance, the expression of FOXP3 seems to be a more consistent marker for functional Treg in mice, than it is in humans [26]–[28]. As for mechanisms of suppression, IL-35 production by Treg is important for suppression in mice [29], while IL-35 is not even expressed by human Treg [30]. Since Treg are currently tested for therapeutic applications in humans, it is especially important to determine to what extend results obtained in mice can be translated to human Treg.

Recently, Pandiyan et al. exemplified a new mechanism of action of Treg in mice, namely their capacity to induce apoptosis in Teff, based on specific cytokine consumption as Treg can consume IL-2 produced by the Teff. Also, addition of IL-2 to co-cultures of Teff and Treg prevented the apoptosis of Teff. Though they did not directly show that addition of IL-2Rγ-chain binding cytokines, which diminished apoptosis, also prevented suppression *in vitro*, *in vivo* they did find that induction of Teff apoptosis is indeed important for Treg function. Furthermore, previous reports show that suppression *in vitro* by murine Treg is prevented by addition of IL-2Rγ-chain binding cytokines [17]. Our current data show some similarities between the mouse and human system, but also reveal an essential difference between mouse and human Tregs; human Treg function is not mediated by apoptosis of Teff. Obviously, human experiments such as these are restricted to *in vitro* assays, and only limited numbers of cells are available. However, *in vitro* Treg assays, similar to those used for mice, can be performed with human cells as well and compared to data obtained in experimental models.

Similar to mice, we show that naturally occurring human Treg very efficiently suppress both proliferation and cytokine production by effector T cells, which can be reversed by addition of IL-2Rγ-chain binding cytokines. These results are consistent with earlier reports on human and murine Treg which show both inhibition of Teff IL-2 mRNA production, as well as Teff proliferation by Treg, and a decrease of suppression of Teff proliferation by addition of high levels of exogenous IL-2 [17], [31], [32]. Also, Treg derived from a highly inflammatory environment, synovial fluid from the joints of JIA patients, suppress Teff proliferation and cytokine production. Obviously, mouse splenocytes differ in many aspects from human PBMC [33]. Here we show that human Teff seem to be less prone to apoptosis than mouse Teff. When comparing cell death in cultures with only Teff, human Teff show hardly any apoptosis (2%), whereas mouse Teff show a higher level of apoptotic cells (20%) [18]. And, importantly, we show that suppression by human Treg does not involve induction of apoptosis in Teff: the absolute numbers of apoptotic cells decrease in the presence of Treg.

IL-2 is an important cytokine for Treg function, both in mice and humans. However, we do not find a decrease of apoptosis in Teff upon addition of IL-2. This may again be due to the low level of apoptosis in Teff in general. However, it could also be explained by the fact that Teff do not necessarily require IL-2 to survive or become activated. This is confirmed by recent data obtained by *in vitro* tests on peripheral blood cells from a specific group of IPEX patients. In these patients Teff produce only low levels of IL-2 and, remarkably, the deficit in Treg function can be overcome by addition of IL-2 to cell cultures. Thus, the *in vivo* lack of Treg function could be explained by the decreased production of IL-2 by Teff in these IPEX patients [3], [34].Altogether, this suggests that in humans IL-2 is very important for Treg function, but is not required for Teff survival and function, as these Teff, despite low IL-2 production, are still highly activated and causing disease.

We show here, in line with earlier publications, that addition of IL-2 and IL-7 abrogates suppression of both Teff proliferation and cytokine production. This could be due to a higher activation of the Teff, as the Teff cultured alone proliferate more and produce more cytokines in the presence of IL-2 and IL-7, or, in case of IL-2, to abrogation of Treg anergy. In addition, we do not find a decrease of the added IL-2 in these cultures with Treg present. This suggests that IL-2 is not consumed by the Treg, although we can not exclude that the level of exogenous IL-2 is simply too high to detect consumption by Treg.

In conclusion, we here point out an important difference between human and murine Treg function: human Treg do not induce apoptosis in Teff to achieve suppression. With these data we emphasize that experimental data from mouse models should be carefully validated in human cells to identify discrepancies, and to ensure that further therapeutic applications are efficient and safe. This does not mean that Treg are less valuable targets for intervention. It could even be argued that if human Treg, instead of eliminating Teff by inducing apoptosis, render Teff either anergic, or even turn them into suppressor cells themselves [35], [36], may be able to exert a stronger bystander suppression in an ongoing inflammatory response.

Our functional data here support that Treg are excellent clinical targets to counteract autoimmune diseases. For optimal functional outcome in human clinical trials, future work should focus on the ability of Treg to suppress proliferation and cytokine production of Teff, rather than induction of Teff apoptosis.

## Materials and Methods

### Ethics statement

This study was conducted according to the principles expressed in the Declaration of Helsinki. The study was approved by the Institutional Review Board of the UMC Utrecht. All patients provided written informed consent for the collection of samples and subsequent analysis.

### Cells, medium and reagents

Peripheral blood mononuclear cells (PBMC) were isolated from peripheral blood of healthy volunteers and JIA patients and synovial fluid mononuclear cells (SFMC) from the synovial fluid of JIA patients, after informed consent, using Ficoll Isopaque density gradient centrifugation (Amersham Biosciences, NJ, USA). RPMI 1640 containing 10 mM HEPES (Seromed), 2 mM L-glutamine 100 U/ml penicillin-streptomycin and 10% human AB serum was used as culture medium (all Invitrogen, Carlsbad, USA). Where indicated, IL-2 (1000 U/ml ( = 60 ng/ml), Chiron, Uxbridge, UK) or IL-7 (10 ng/ml, PeproTech Inc, Rocky Hill, NJ, USA), were added.

### Suppression assay

CD4^+^ CD25^−^ effector T cells (Teff), were magnetically isolated from PBMC using a CD4 T Lymphocyte Enrichment Set (BD Biosciences). Subsequently, CD25^+^ T cells were depleted using CD25 Magnetic Particles (BD Biosciences). All magnetic cell isolations were performed according to the manufacturer's instructions. The CD4^+^CD25^−^ T cells were labeled with 3 µM CFSE for 10 min at 37°C and extensively washed. 25,000 Teff (Teff) were plated into anti-CD3-coated wells (OKT-3, 1.5 µg/ml), and to control for higher cell numbers in co-cultures (crowdedness) 50,000 Teff were plated (Teff+Teff). CD4^+^CD25^+^CD127^−^ T cells were sorted as Treg from PBMC [37], [38] (with an average of 58% FOXP3^+^ cells±13% s.d.) or SFMC [39] (with an average of 24% FOXP3^+^ cells±12% s.d.) by FACS Aria (BD Biosciences) and added in different ratios to Teff. T cell depleted, irradiated autologous PBMC (3500 Rad) were used as Antigen presenting cells (APC), 30,000 per well. Cells were cultured for 5 days and proliferation was measured by flow cytometry on a FACS Calibur (BD Biosciences). The levels of FOXP3^+^ cells in the CD4^+^CD25^+^CD127^low^ T cells directly isolated from PBMC or SFMC were lower than expected. This is due to an underestimation of the percentage of FOXP3^+^ cells (See Figure S4). All data were analyzed using Cellquest software.

### Flow cytometry staining

To determine levels of apoptosis, cells were stained with Annexin V PE and 7-AAD, using a staining kit according to the manufacturer's instructions (all BD Biosciences). CFSE^+^ cells were gated to determine cell death within the Teff population. For FOXP3 analysis, PBMC were washed twice in FACS buffer (PBS containing 2% FCS and 0.1% sodium azide), adjusted to 0.5−1×10^6^ cells/ml in FACS buffer and blocked with mouse serum (5 min at 4°C). Subsequently, the cells were incubated in 50 µl FACS buffer containing three appropriately diluted PE, FITC or PerCP labeled mAbs against human CD4 (clone RPA-T4), CD25 (clone 2A3), CD127 (clone hIL-7R-m21), all from BD Biosciences. For intranuclear staining of APC or Pacific Blue FOXP3 (clone PCH101), V450 FOXP3 (clone 259D, BD Bioscience) or Isotype Control, the cells were first surface stained, then fixed, permeabilized and stained using the FOXP3 staining kit (eBioscience) according to the manufacturer's instructions. Cells were analyzed on a FACS Calibur (BD Biosciences). All data were analyzed using Cellquest software.

### Analysis of cytokine production by multiplexed particle-based flow cytometric assay

Cell culture supernatants were collected, stored at −80°C and processed within 1 month. Cytokine concentrations were measured with the Bio-Plex system in combination with the Bio-Plex Manager software, version 4.0 (Bio-Rad Laboratories, Hercules, CA, USA), which employs the Luminex xMAP technology as previously described [40]. The following cytokines were measured: IL-2, IL-5, IL-7, IL-10, IL-13, IL-17, tumor necrosis factor-α (TNF-α), and interferon-γ (IFNγ).

### Statistical analysis

For statistical analysis of multiple groups One-way ANOVA or nonparametric ANOVA; Kruskal-Wallis test, were used. Bonferroni or Dunn's Multiple Comparison Test post test were used, to compare between 2 selected groups. To compare between two groups, non-parametric T-test, Mann Whitney was used. P values below 0.05 were considered significant.

## Supporting Information

Table S1 IL-2 and IL-7 inhibit nTreg mediated suppression of Teff cytokine production, but nTreg do not consume IL-2 or IL-7.
(0.06 MB PDF)

Table S2 SF-Treg suppress Teff cytokine production.
(0.03 MB PDF)

Table S3 The level of cytokine suppression for nTreg and SF-Treg.
(0.01 MB PDF)

Figure S1 Proliferation and apoptosis of Teff after 3, 4, and 5 days of culture in the presence and absence of Treg. (A) Gated CFSE+ Teff in the presence of APC (left plot) or APC + Treg (middle plot) after 5 days of culture. For comparison, Treg only + APC are shown as well (right plot). 1 representative example of n = 9. (B) Gated CFSE+ Teff in the presence of APC (left plot) or APC + Treg (right plot) after 3 days of culture. For comparison, Treg only + APC are shown as well (right plot). 1 representative example of n = 3. (C) Average percentage, and (D) absolute number (corrected for cell input) of apoptotic Teff, expressing 7-AAD and Annexin V, for several co-culture ratios of Teff + Treg, Teff + Teff and Teff alone after 3 (black bars), 4 (white bars) or 5 days (grey bars) of culture (n = 3). Error bars represent means Â± s.e.m.
(1.66 MB TIF)

Figure S2 Sheath Fluid, containing ethanol, dose dependently induces apoptosis in Teff. (A) Percentage of apoptotic Teff, expressing 7-AAD and Annexin V, after culture for 5 days without (left panel) or with increasing amounts of PBS as a control (middle panel) or Sheath Fluid to induce apoptotic cells (right panel). 1 representative of n = 2. (B) Average percentage of apoptotic Teff, expressing 7-AAD and Annexin V, for increasing concentrations of PBS (white bars) and Sheath Fluid (black bars) (n = 2). Error bars represent means Â± s.e.m.
(1.08 MB TIF)

Figure S3 Annexin V expressing Teff and apoptotic Teff, expressing both Annexin V and 7-AAD, in the presence and absence of IL-2 and IL-7. (A) Annexin V expression of gated CFSE+ Teff cultured for 5 days without additional stimuli (left panel), in the presence of 20% Sheath Fluid to induce apoptotic cells (middle panel), or in the presence of IL-2 or IL-7 (right panel). 1 representative example for each condition is shown. (B) Percentage of apoptotic Teff, expressing 7-AAD and Annexin V, alone or in the presence of Treg (1-1) in the absence (left panel) or presence of IL-2 (middle panel) or IL-7 (right panel). 1 representative example of n = 5.
(1.63 MB TIF)

Figure S4 Percentage of FOXP3 expressing cells within the CD4+CD25+CD127low Treg population. (A) CD25 and CD127 expression of gated CD4+ T cells. The gate used for sorting the CD4+CD25+CD127low Treg population is indicated. For comparison of FOXP3 expression CD4+CD25- cells were gated. 1 representative example is shown. (B) FOXP3 expression measured by different FOXP3 antibodies within the CD4+CD25+CD127 low Treg population (left panel), within the CD4+CD25- cells (middle panel), and corresponding isotype controls gated on CD4+CD25+CD127low Treg (right panel). 1 representative example of n = 4.
(0.91 MB TIF)

Figure S5 Annexin V and 7-AAD expressing cells and apoptotic Teff, expressing both Annexin V and 7-AAD, in the presence and absence of Treg. (A) Total cells expressing AnnexinV (left panel) and 7-AAD (right panel), in the presence and absence of Treg at day 5 of culture. 1 representative example of n = 9. (B) Total cells expressing AnnexinV (left panel) and 7-AAD (right panel), in the presence and absence of Treg at day 3 of culture. 1 representative example of n = 3. (C) Average absolute number of apoptotic Teff, expressing 7-AAD and Annexin per cell division (0 = undivided cells), in the absence (black bars) and presence (white bars) of Treg at day 5 of culture. (n = 4) Error bars represent means Â± s.e.m.
(1.97 MB TIF)
