# Supplementary material for: Human Regulatory T Cell Suppressive Function Is Independent of Apoptosis Induction in Activated Effector T Cells
Source: PLoS One. 2009 Sep 25;4(9):e7183. doi: 10.1371/journal.pone.0007183 (PMC2746309; doi:10.1371/journal.pone.0007183)
Supplement: Table S1 — IL-2 and IL-7 inhibit nTreg mediated suppression of Teff cytokine production, but nTreg do not consume IL-2 or IL-7. (0.06 MB PDF) [file pone.0007183.s001.pdf]

Condition	Culture	IL-2	IL-7	IL-5	IL-13	IL-10	IFNγ	TNFα	IL-17
-	Teff	30.7 (45.1)	0.0 (0.0)	14.6 (14.3)	112.3 (99.6)	141.4 (192.2)	367.8 (340.1)	461.2 (403.6)	773.3 (856.9)
-	Teff+ Teff	78.3 (203.1)	0.0 (0.0)	26.7 (26.8)	142.8 (133.4)	286.4 (363.8)	828.3 (745.2)	486.8 (548.0)	2273.6 (2859.2)
-	10-1	7.4 (5.3)	0.0 (0.0)	14.9 (12.3)	40.2 (35.8)	46.7 (44.8)	253.6 (324.4)	232.8 (319.6)	698.6 (847.7)
-	5-1	2.9 (2.5)	0.0 (0.0)	14.4 (8.8)	27.2 (25.7)	59.3 (52.1)	131.2 (118.3)	202.6 (290.1)	721.4 (721.7)
-	2-1	2.1$^{\#}$ (1.7)	0.0 (0.0)	9.1 (8.8)	13.3* (13.2)	59.8 (36.2)	17.9 (20.7)	110.5 (198.7)	393.5 (314.9)
-	1-1	3.5$^{\#}$ (6.7)	0.0 (0.0)	5.6 (4.0)	10.1* (7.7)	61.3 (40.5)	10.9* (13.7)	32.1 (51.3)	844.2 (973.0)
-	nTreg	0.6 (1.0)	0.0 (0.0)	2.2 (0.3)	0.0 (0.0)	12.3 (5.3)	0.0* (0.0)	2.7 (3.4)	44.4 (28.4)
		IL-2$^{\#}$	IL-7^{ns}	IL-5$^{\$}$	IL-13$^{\$}$	IL-10*	IFNγ^{ns}	TNFα$^{\#}$	IL-17$^{\#}$
+IL-2	Teff	12878.2 (2170.6)	0.0 (0.0)	360.3 (177.5)	4034.3 (1244.5)	2356.2 (2832.7)	1036.7 (513.5)	1270.8 (560.8)	8328.4 (5514.3)
+IL-2	Teff+ Teff	8756.7 (2776.0)	0.0 (0.0)	267.6 (168.8)	2811.4 (1054.6)	1853.1 (594.7)	1478.4 (806.8)	1365.7 (985.3)	12036.4 (4362.6)
+IL-2	10-1	13720.8 (4420.5)	0.0 (0.0)	298.7 (150.3)	4459.9 (262.8)	860.6 (822.5)	791.3 (122.1)	1022.6 (1262.0)	9417.6 (4929.7)
+IL-2	5-1	11399.4 (3157.0)	0.0 (0.0)	455.1 (170.7)	3511.6 (1497.2)	885.9 (318.3)	893.5 (353.7)	697.4 (881.1)	6805.1 (5100.3)
+IL-2	2-1	11642.7 (6578.4)	0.0 (0.0)	625.5 (364.7)	3082.3 (612.3)	1241.2 (973.0)	663.5 (223.4)	745.1 (419.3)	10086.6 (4898.7)
+IL-2	1-1	6494.2 (7442.3)	0.0 (0.0)	394.5 (412.2)	1927.7 (1478.0)	2005.5 (1467.8)	513.6 (426.8)	509.0 (442.6)	9473.2 (6655.3)
+IL-2	nTreg	13213.0 (2457.5)	0.0 (0.0)	225.0 (184.6)	329.7$^{\#}$ (159.2)	2214.1 (683.9)	26.0 (4.3)	49.6 (6.4)	5922.7 (1980.1)
		IL-2^{ns}	IL-7$^{\$}$	IL-5^{ns}	IL-13^{ns}	IL-10$^{\#}$	IFNγ^{ns}	TNFα^{ns}	IL-17^{ns}
+IL-7	Teff	52.0 (94.1)	2245.8 (417.7)	32.6 (11.0)	373.5 (103.5)	2114.2 (1872.5)	1126.4 (1042.2)	778.4 (293.3)	4869.1 (3014.3)
+IL-7	Teff+ Teff	2.5 (0.8)	983.3$^{\#}$ (127.1)	43.2 (14.4)	334.7 (176.4)	1854.1 (1233.8)	1486.3 (887.6)	906.4 (345.3)	5021.8 (2421.1)
+IL-7	10-1	2.2 (0.7)	2048.7 (557.2)	62.9 (36.9)	403.1 (100.4)	1708.6 (1795.6)	1131.3 (827.4)	662.3 (391.0)	4210.7 (2207.8)
+IL-7	5-1	3.0 (0.2)	2103.1 (257.6)	45.5 (34.0)	331.2 (56.9)	2049.0 (1996.1)	1205.4 (1213.2)	794.1 (286.4)	5026.6 (1879.6)
+IL-7	2-1	3.9 (5.3)	2479.4 (242.1)	62.8 (36.1)	282.7 (81.0)	861.4 (507.6)	570.7 (462.5)	621.3 (227.6)	3822.0 (867.8)
+IL-7	1-1	5.1 (5.4)	3427.8$^{\#}$ (655.3)	86.5 (85.1)	354.1 (128.4)	719.3 (430.3)	342.9 (305.5)	375.0 (117.4)	4396.8 (2152.9)
+IL-7	nTreg	1.2 (0.8)	4590.8$^{\$}$ (77.1)	13.8 (7.4)	38.4 (17.0)	203.3 (53.4)	2.6 (0.6)	20.5* (2.6)	399.5 (152.2)

Supplementary Table 1. IL-2 and IL-7 inhibit nTreg mediated suppression of Teff cytokine production, but nTreg do not consume IL-2 or IL-7. Cells were culture in 75 μl medium for 5 days. Mean levels of cytokines, present in culture medium on day 5 of culture, in medium (-)(N=9), with addition of IL2 (IL-2)(N=5) or with addition of IL7 (IL-7)(N=5). All values (mean (SD)) are expressed in pg/ml.

When values were compared to Teff only: * $p<0.05$, # $p<0.01$, \$ $p<0.001$ (see table above per culture condition).
When cytokine values in cultures +IL-2 or +IL-7 were compared to medium: * $p<0.05$, # $p<0.01$, \$ $p<0.001$ ns=non-significant (see upper row of tables +IL-2 or +IL-7, significance is depicted per cytokine)
