# Supplementary material for: Human Regulatory T Cell Suppressive Function Is Independent of Apoptosis Induction in Activated Effector T Cells
Source: PLoS One. 2009 Sep 25;4(9):e7183. doi: 10.1371/journal.pone.0007183 (PMC2746309; doi:10.1371/journal.pone.0007183)
Supplement: Table S2 — SF-Treg suppress Teff cytokine production. (0.03 MB PDF) [file pone.0007183.s002.pdf]

IL-2	IL-7	IL-5	IL-13	IL-10	IFNγ	TNFα	IL-17	
0.8 (0.9)	0.0 (0.0)	38.9 (38.1)	153.9 (130.3)	210.7 (168.3)	1372.5 (952.8)	380.7 (231.1)	2472.5 (1344.4)	Teff
0.6 (0.5)	0.0 (0.0)	91.5 (86.8)	284.6 (227.2)	357.9 (258.6)	1777.5 (933.6)	535.3 (166.50	3626.2 (1693.8)	Teff+ Teff
0.1 (0.1)	0.0 (0.0)	22.0 (21.4)	140.0 (189.4)	134.1 (81.9)	818.1 (1006.0)	244.5 (287.8)	1257.0 (877.1)	10-1
0.3 (0.6)	0.0 (0.0)	20.3 (17.7)	102.1 (115.5)	104.9 (58.8)	676.4 (1001.0)	202.8 (292.7)	1573.5 (1152.0)	5-1
0.1 (0.1)	0.0 (0.0)	4.8 (5.0)	22.8 (22.2)	83.9 (40.7)	383.7 (638.7)	123.5 (198.7)	940.6 (688.7)	2-1
0.0 (0.1)	0.0 (0.0)	1.3 (2.3)	7.3 (9.5)	70.0 (42.8)	105.8 (183.2)	35.0 (59.6)	487.4 (475.1)	1-1
0.0 (0.0)	0.0 (0.0)	1.5 (2.5)	3.0 (5.1)	32.0 (24.4)	3.4 (5.9)	2.4 (4.2)	96.3 (143.6)	nTreg

-

Supplementary Table 2. SF-Treg suppress Teff cytokine production. Cells were cultured in 75 μl medium for 5 days (N=3). Mean levels of cytokines, present in culture medium on day 5 of culture. All values (mean (SD)) are expressed in pg/ml.
