# Supplementary material for: Human Regulatory T Cell Suppressive Function Is Independent of Apoptosis Induction in Activated Effector T Cells
Source: PLoS One. 2009 Sep 25;4(9):e7183. doi: 10.1371/journal.pone.0007183 (PMC2746309; doi:10.1371/journal.pone.0007183)
Supplement: Table S3 — The level of cytokine suppression for nTreg and SF-Treg. (0.01 MB PDF) [file pone.0007183.s003.pdf]

nTreg level of suppression

IL-5	IL-13	IL-10	IFNg	TNFa	IL-17	
0,0	0,0	0,0	0,0	0,0	0,0	Teff
-158,6#	-32,3	-5940,3	-275,1	-132065,5	-74842,6	Teff+Teff
(209,8)	(62,2)	(16939,9)	(416,4)	(396125,5)	(223559,2)	
-209,8	57,1	-5260,9	64,1	-2939,6	-64657,3*	10_1
(346,7)	(37,2)	(13951,6)	(36,1)	(7919,8)	(170978,5)	
-221,8	73,2	-1958,2	78,9	57,2	-91260,8	5_1
(258,1)	(15,7)	(4821,8)	(18,1)	(34,4)	(223069,9)	
-95,6	78,4	-14056,6	96,2	-2043,2	-57914,0	2_1
(224,5)	(29,5)	(39414,3)	(4,8)	(6003,1)	(163598,7)	
10,2	87,2	-14488,1	96,3	83,0	-80248,3*	1_1
(94,4)	(11,5)	(43205,0)	(6,2)	(32,7)	(240507,0)	

SF-Treg level of suppression

IL-5	IL-13	IL-10	IFNg	TNFa	IL-17	
0,0	0,0	0,0	0,0	0,0	0,0	Teff
-111,2#	-124,5	-111,2	-44,8	-73,4	-59,0	Teff+Teff
(98,6)	(71,5)	(98,6)	(37,1)	(90,5)	(61,4)	
23,2	43,5	23,2	57,4	52,1	52,2*	10_1
(26,8)	(70,3)	(26,8)	(36,8)	(41,7)	(29,3)	
33,9	57,2	33,9	68,3	63,3	43,1	5_1
(31,1)	(43,2)	(31,1)	(39,1)	(45,5)	(32,5)	
38,5	90,1	38,5	83,5	78,4	65,4	2_1
(46,3)	(8,7)	(46,3)	(26,3)	(31,7)	(22,7)	
50,2	97,0	50,2	95,6	94,0	81,0*	1_1
(37,7)	(3,5)	(37,7)	(7,7)	(9,7)	(19,9)	

Supplementary Table 3. The level of cytokine suppression for nTreg and SF-Treg. In this table we show the averages (SD) level of suppression of cytokines in the cultures. We compared per cytokine, per co-culture condition whether there was a significant difference in cytokine suppression between nTreg and SF-Treg, by Mann Whitney U test, * $p< 0.05$, # $p<0.01$.
