# Supplementary figures and images for: Human Regulatory T Cell Suppressive Function Is Independent of Apoptosis Induction in Activated Effector T Cells
Source: PLoS One. 2009 Sep 25;4(9):e7183. doi: 10.1371/journal.pone.0007183 (PMC2746309; doi:10.1371/journal.pone.0007183)

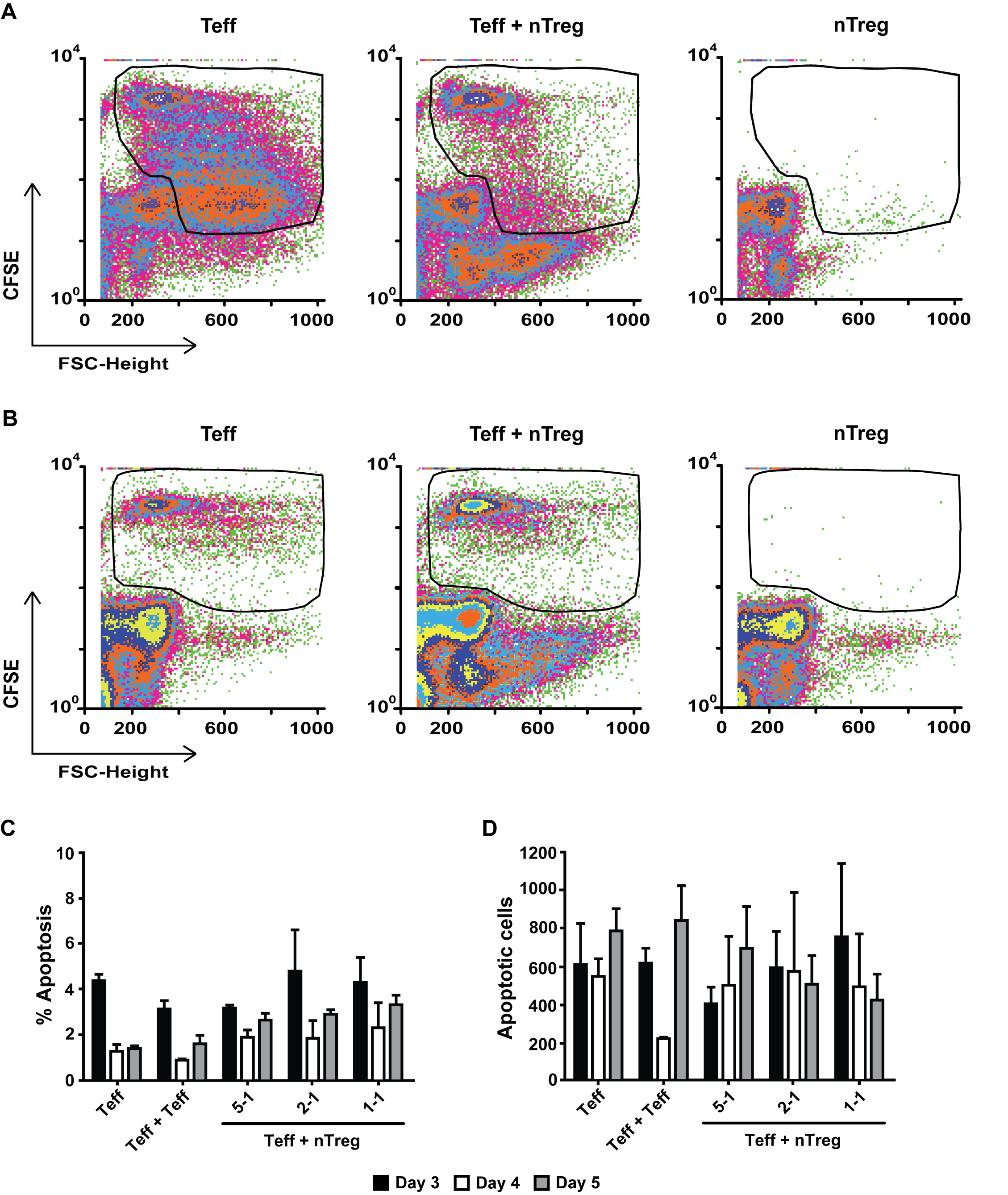

Supplement: Figure S1 — Proliferation and apoptosis of Teff after 3, 4, and 5 days of culture in the presence and absence of Treg. (A) Gated CFSE+ Teff in the presence of APC (left plot) or APC + Treg (middle plot) after 5 days of culture. For comparison, Treg only + APC are shown as well (right plot). 1 representative example of n = 9. (B) Gated CFSE+ Teff in the presence of APC (left plot) or APC + Treg (right plot) after 3 days of culture. For comparison, Treg only + APC are shown as well (right plot). 1 representative example of n = 3. (C) Average percentage, and (D) absolute number (corrected for cell input) of apoptotic Teff, expressing 7-AAD and Annexin V, for several co-culture ratios of Teff + Treg, Teff + Teff and Teff alone after 3 (black bars), 4 (white bars) or 5 days (grey bars) of culture (n = 3). Error bars represent means Â± s.e.m. (1.66 MB TIF) [file pone.0007183.s004.tif]

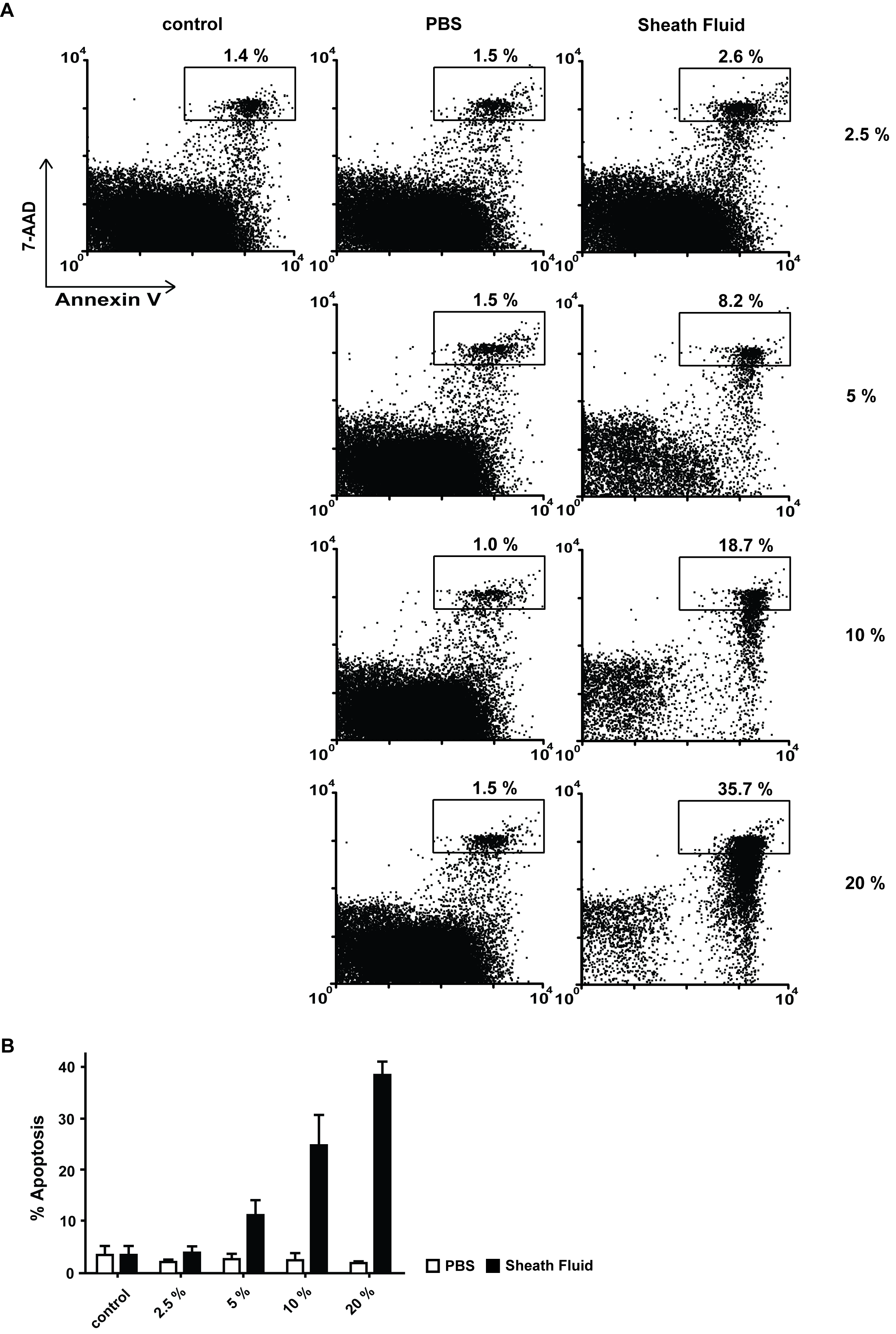

Supplement: Figure S2 — Sheath Fluid, containing ethanol, dose dependently induces apoptosis in Teff. (A) Percentage of apoptotic Teff, expressing 7-AAD and Annexin V, after culture for 5 days without (left panel) or with increasing amounts of PBS as a control (middle panel) or Sheath Fluid to induce apoptotic cells (right panel). 1 representative of n = 2. (B) Average percentage of apoptotic Teff, expressing 7-AAD and Annexin V, for increasing concentrations of PBS (white bars) and Sheath Fluid (black bars) (n = 2). Error bars represent means Â± s.e.m. (1.08 MB TIF) [file pone.0007183.s005.tif]

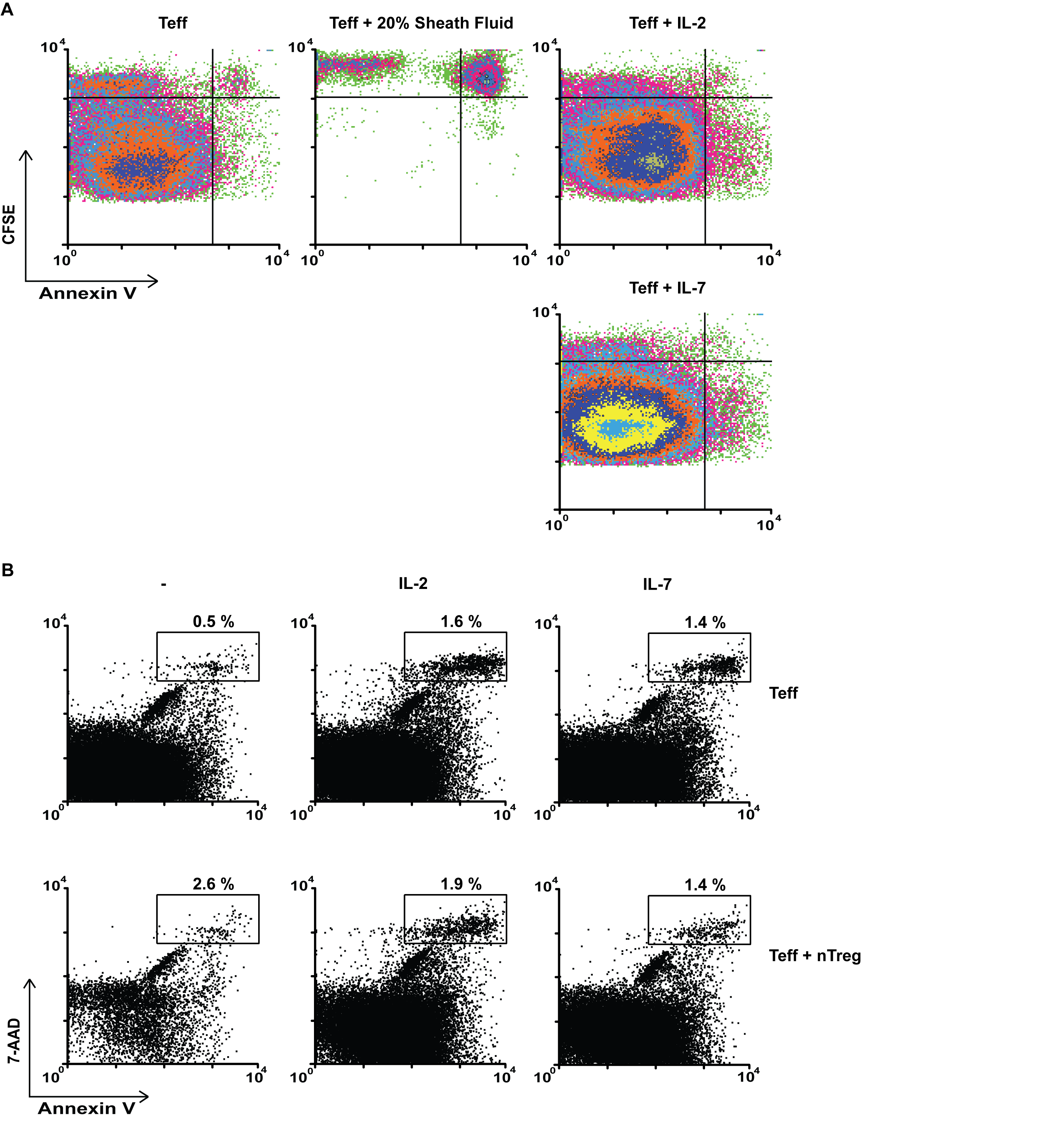

Supplement: Figure S3 — Annexin V expressing Teff and apoptotic Teff, expressing both Annexin V and 7-AAD, in the presence and absence of IL-2 and IL-7. (A) Annexin V expression of gated CFSE+ Teff cultured for 5 days without additional stimuli (left panel), in the presence of 20% Sheath Fluid to induce apoptotic cells (middle panel), or in the presence of IL-2 or IL-7 (right panel). 1 representative example for each condition is shown. (B) Percentage of apoptotic Teff, expressing 7-AAD and Annexin V, alone or in the presence of Treg (1-1) in the absence (left panel) or presence of IL-2 (middle panel) or IL-7 (right panel). 1 representative example of n = 5. (1.63 MB TIF) [file pone.0007183.s006.tif]

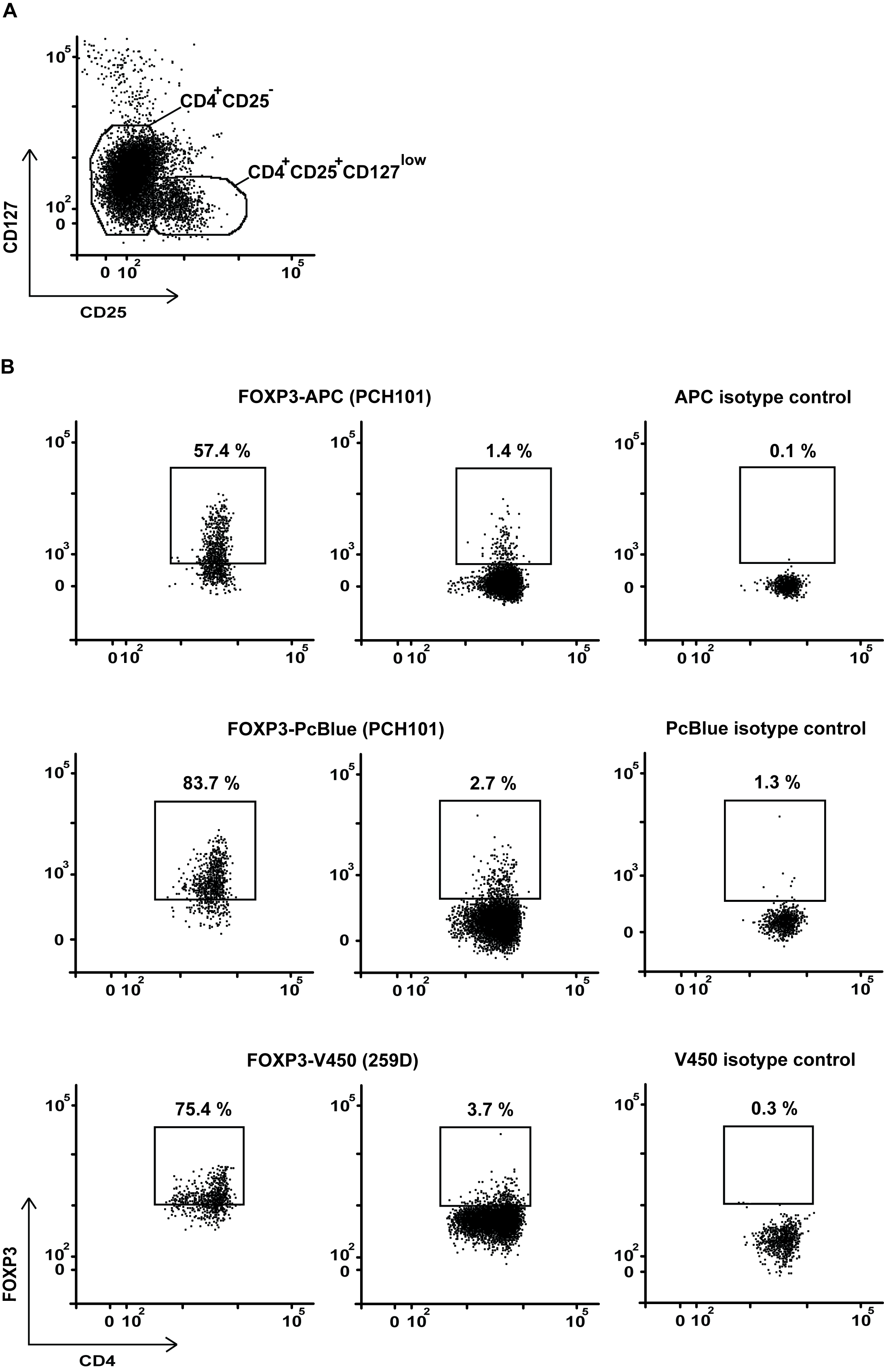

Supplement: Figure S4 — Percentage of FOXP3 expressing cells within the CD4+CD25+CD127low Treg population. (A) CD25 and CD127 expression of gated CD4+ T cells. The gate used for sorting the CD4+CD25+CD127low Treg population is indicated. For comparison of FOXP3 expression CD4+CD25- cells were gated. 1 representative example is shown. (B) FOXP3 expression measured by different FOXP3 antibodies within the CD4+CD25+CD127 low Treg population (left panel), within the CD4+CD25- cells (middle panel), and corresponding isotype controls gated on CD4+CD25+CD127low Treg (right panel). 1 representative example of n = 4. (0.91 MB TIF) [file pone.0007183.s007.tif]

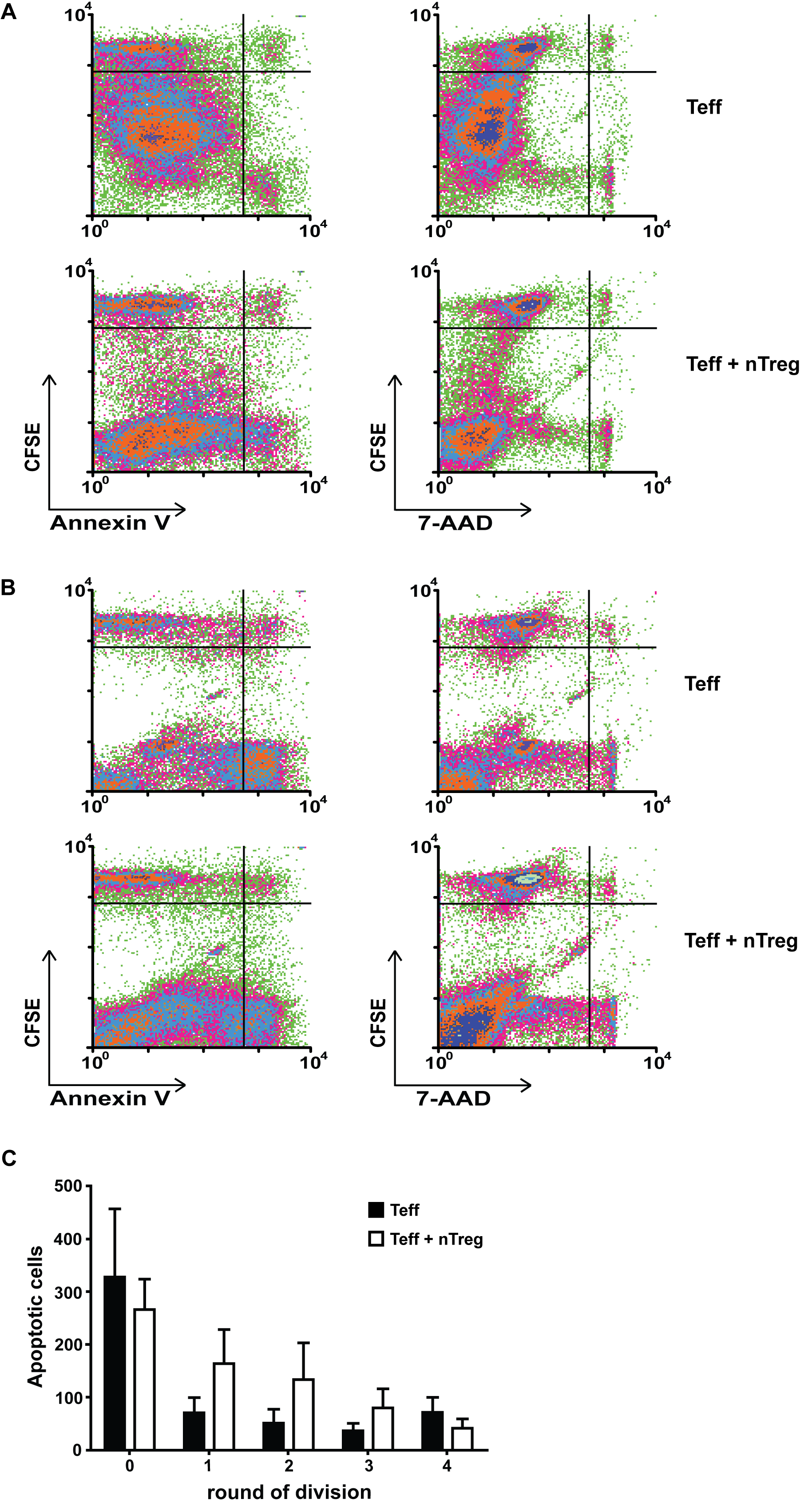

Supplement: Figure S5 — Annexin V and 7-AAD expressing cells and apoptotic Teff, expressing both Annexin V and 7-AAD, in the presence and absence of Treg. (A) Total cells expressing AnnexinV (left panel) and 7-AAD (right panel), in the presence and absence of Treg at day 5 of culture. 1 representative example of n = 9. (B) Total cells expressing AnnexinV (left panel) and 7-AAD (right panel), in the presence and absence of Treg at day 3 of culture. 1 representative example of n = 3. (C) Average absolute number of apoptotic Teff, expressing 7-AAD and Annexin per cell division (0 = undivided cells), in the absence (black bars) and presence (white bars) of Treg at day 5 of culture. (n = 4) Error bars represent means Â± s.e.m. (1.97 MB TIF) [file pone.0007183.s008.tif]
